# Key Odorants from Pig Production Based on Improved Measurements of Odor Threshold Values Combining Olfactometry and Proton-Transfer-Reaction Mass Spectrometry (PTR-MS)

**DOI:** 10.3390/s18030788

**Published:** 2018-03-06

**Authors:** Michael Jørgen Hansen, Pernille Lund Kasper, Anders Peter S. Adamsen, Anders Feilberg

**Affiliations:** 1 Department of Engineering, Aarhus University, 8000 Aarhus, Denmark; peka@eng.au.dk (P.L.K.); af@eng.au.dk (A.F.); 2 SEGES, 8200 Aarhus N, Denmark; apa@seges.dk

**Keywords:** odor threshold values, odor activity value, odorants, odor, pigs

## Abstract

Analytical measurements of odorants in combination with odor threshold values is an alternative to sensory measurements that can be used to evaluate abatement technologies for pig production facilities. The purpose of the present study was to estimate odor threshold values for key odorants found in pig house air. A new method was applied where an olfactometer was used to dilute the sample air and the concentrations of odorants presented to the panelists at the dilutions steps were measured by proton-transfer-reaction mass spectrometry (PTR-MS). The results demonstrate that the odor threshold values of acetic acid, butanoic acid, and 4-methylphenol are considerably lower than reported previously, whereas the values of hydrogen sulfide, methanethiol and dimethylsulfide were comparable. Consequently, acetic acid, butanoic acid, and 4-methyl-phenol will have a larger influence on odor from pig production facilities than previously assumed. The results highlight the necessity for directly measuring exposure concentrations when determining odor threshold values.

## 1. Introduction

Odor from agricultural facilities measured by a chemical method has often been suggested as an alternative to the olfactometric method [1], which is based on dilution-to-threshold with human panelists. The olfactometric method has several drawbacks such as impaired recovery of odorants during storage in sample bags [2,3,4,5] and the dilution system in the olfactometer [6,7] as well as a high variability due to the use of human panelists [8]. Different methods can be applied for measuring odorants from agricultural facilities including on-line mass spectrometry [9,10,11,12] and laboratory based gas chromatographic methods [13,14,15]. However, the applicability of the chemical methods in relation to agricultural facilities and odor abatement technologies (e.g., air cleaning) requires that the chemical measurements can be converted into an odor concentration or nuisance level. Recently, Wu et al. [16] demonstrated the applicability of odor threshold values for predicting odor concentration for odorant mixtures. In a simple approach, the odor activity value is calculated as the gas-phase concentrations divided by the odor threshold values. The odorant contributions are assumed to be additive and the calculated odor concentration is denoted “Sum of odor activity values” (SOAV). This approach gave a reasonable relationship with the odor concentration measured by olfactometry although the absolute odor concentration was underestimated [16]. Significantly improved results were obtained by the so-called “Equivalent odor concentration” (EOC), which takes into account the slope of the odor intensity versus the odor concentration for the individual odorants [16]. On a relative scale, it was recently demonstrated that SOAV could be used to evaluate odor abatement technologies [17]. The advantages of using chemical concentration measurements of odor concentration include highly improved intra/inter-laboratory precision and a potentially insignificant influence of sampling and sample storage compared to olfactometry. However, the methods are completely dependent on accurately assessed odor threshold values for the individual odorants. Odor threshold values found in literature demonstrate large variations [18] and there is a need for more precise and standardized estimates. In exhaust air from pig production facilities a number of odorants can be identified including carboxylic acids, sulfur compounds, amines, ketones, aldehydes, phenols and indoles. It has previously been indicated that hydrogen sulfide, methanethiol, trimethylamine and 4-methyl-phenol are key odorants in pig house air [19,20]. Carboxylic acids (e.g., acetic and butanoic acid) are some of the odorants found at the highest concentrations in air from pig houses [10,21,22], but previously they have not been considered as key odorants due to relatively high odor threshold values [18]. However, recent studies [23,24,25,26,27,28,29] have found lower odor threshold values of carboxylic acids than previously assumed. Consequently, there is a need to examine the odor threshold values of key odorants found in pig house air in order to improve the evaluation of odor nuisance from pig production based on chemical measurements. In this work, a new method for measuring odor threshold values is presented and applied. The normal approach is that the concentrations of the odorants during exposure are estimated indirectly based on input concentration and the dilution factors for the olfactometer. This may result in large systematic errors due to impaired recovery of odorants [6,7]. The new method is based on dilution-to-threshold olfactometry in combination with accurate and time-resolved measurement of the odorant concentration during exposure by means of proton-transfer-reaction mass spectrometry (PTR-MS). The application of PTR-MS for measuring odorant exposure concentration for this purpose is demonstrated here for the first time. The aim of the present study was to estimate odor threshold values for key odorants normally found in pig house air based on new and improved measurements.

## 2. Materials and Methods 

### 2.1. Selection of Panelists 

Prior to the experiment human panelists were selected based on their sensitivity to n-butanol as described by the European standard for olfactometry [1]. A group of 18 persons was tested on three individual days with at least one day between using an olfactometer and 24–30 threshold measurements were performed on n-butanol for each person. Based on these threshold measurements a group of eight panelists was selected. The panelists consisted of three men and five women with an average age of 31 years. The use of human panelists in the current work complies with the Declaration of Helsinki for Medical Research involving Human Subjects. The work has been approved by the Steering Committee of the project (Project No. 3405-11-0302) by oral consent.

### 2.2. Measurement of Odor Threshold Values

The odorants included in the odor threshold measurements were hydrogen sulfide, methanethiol, dimethyl sulfide, acetic acid, butanoic acid, and 4-methylphenol. Due to the previously demonstrated time delay of some odorants in the olfactometer [6,7] the presentation time at each dilution step was set at 2.2 s (average time for one breath) for hydrogen sulfide, methanethiol, and dimethyl sulfide and 6.6 s (average time for three breaths) for acetic acid, butanoic acid, and 4-methylphenol. The measurements were carried out over ten days and for each odorant, each panelist estimated the odor threshold values at two different days. For hydrogen sulfide, methanethiol, and dimethyl sulfide each panelist estimated the dilution to threshold 12 times giving a maximum of 96 threshold estimates per odorant. For acetic acid, butanoic acid and 4-methylphenol each panelist estimated the dilution to threshold eight times giving a maximum of 64 threshold estimates per odorant. Fewer threshold estimates were conducted on the latter group of odorants to avoid olfactory fatigue of the panelists due to the longer presentation time. 

### 2.3. Odorants 

Hydrogen sulfide, methanethiol (both from AGA, Copenhagen, Denmark) and dimethyl sulfide (Air Liquide, Horsens, Denmark) were introduced from certified gas cylinders (5 ppm_v_ in nitrogen). Dimethyl sulfide was introduced without predilution, whereas hydrogen sulfide and methanethiol were prediluted with synthetic air (AGA, Copenhagen, Denmark). Acetic acid, butanoic acid, and 4-methylphenol were introduced from permeation tubes (VICI Metronics, Inc., Houston, TX, USA) using a permeation oven (Dynacalibrator model 150, VICI Metronics Inc., Houston, TX, USA). The gas release (ng min^−1^) was measured gravimetrically with at least three repetitions. The flow through the permeation oven was 300 mL min^−1^ for all three odorants and the temperature was set at 60 °C for acetic acid, 80 °C for butanoic acid, and 100 °C for 4-methylphenol. Acetic acid and butanoic acid were introduced without predilution, whereas 4-methylphenol was prediluted with synthetic air. Mass flow controllers (Bronkhorst, Ruurlo, The Netherlands) were used to control the gas flow from the gas cylinders and for the predilution. The gas delivery system including reduction valves, mass flow controllers and PTFE tubing was allowed to equilibrate for at least one hour before the measurements were performed. 

### 2.4. Analytical Methods 

A TO8 olfactometer (Odournet GmbH, Kiel, Germany) was used to estimate the odor threshold values. The olfactometer was designed for four panelists and was based on the yes-no method. The olfactometer was able to perform dilutions from 65,536 to 4 times dilutions with a step factor of two. The dilutions were presented in ascending order. The dilution air to the olfactometer was provided by a Dr. sonic compressor (Fini, Bologna, Italy) and before entering the olfactometer the dilution air was filtered by a column containing silica gel and activated charcoal. The olfactometer was calibrated by Odournet GmbH according to the European standard for olfactometry [1].

A high sensitivity PTR-MS (Ionicon Analytik GmbH, Innsbruck, Austria) was used to estimate the concentration of odorants delivered to the nose cone of the olfactometer at each dilution step. The drift tube conditions for the PTR-MS were set at a voltage of 600 V, a pressure between 2.1–2.2 mbar and a temperature at 75 °C. The inlet was made by a 1.2 m polyether ether ketone (PEEK) tubing with an outer diameter of 1.6 mm and an inner diameter of 0.064 mm. The inlet temperature was 75 °C and the inlet flow ca. 100 mL·min^−1^. The PTR-MS was calibrated using a one-point calibration with the gas standards listed in Section 2.3. The relative standard deviation on the calibration standards as gas bottles or permeation tubes were between 5–10%. The detection limit was estimated as three times the standard deviation on charcoal-filtered air using a Supelpure™ HC filter (Supelco, Bellefonte, PA, USA), see supplementary information. An ion dwell time of 0.5 s or one second was used in order to measure the odorant concentration at the exact time of exposure after 2.2 and 6.6 s presentation time, respectively. At each measurement day, the actual concentration delivered to the nose cone at each dilution step was estimated based on measurements by PTR-MS after the threshold measurements to avoid disturbance of the panelists. Measurements at each dilution step was repeated four times. The measured concentrations were corrected for the recovery of the odorants in the PTR-MS inlet system at the time of exposure (2.2 and 6.6 s) according to the method described by Kasper et al. [7]. 

### 2.5. Data Analysis

For each panelist at each presentation the dilution-to-threshold was estimated as the geometric mean of the dilution step where the panelist could detect the odorant the first time and the dilution step prior to this. The statistical difference between panelists for threshold estimates (logarithmic values) were analyzed using linear models in SAS. The significance level was defined as a *p*-value below 0.05. 

## 3. Results and Discussion

In the present study, a new method combining an olfactometer and a PTR-MS was applied for measuring odor threshold values for odorants normally found in pig house air. Olfactometers are often used to estimate odor threshold values, but in many cases, the concentrations of chemical odorants at the individual dilution steps are estimated based on the concentrations of the undiluted samples and the dilution factors. However, the olfactometer may have an influence on the recovery of the chemical odorants [6,7] which may lead to overestimation of the odor threshold values. In the new method applied in the present study the fast response and low detection limit of the PTR-MS was used to estimate the concentrations of chemical odorants at the individual dilution steps giving a more precise estimate of the odor threshold values. 

**Figure 1:**
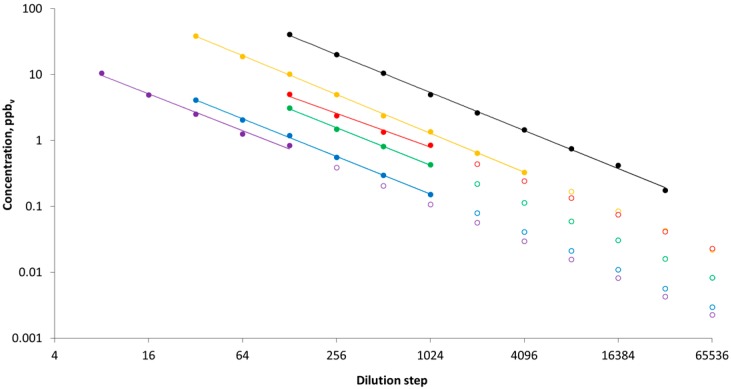
Measured (closed symbols) and estimated (open symbols) concentrations of dimethyl sulfide (black), hydrogen sulfide (yellow), acetic acid (red), butanoic acid (green), methanethiol (blue), 4-methylphenol (purple), as a function of dilution step. Estimated concentrations are calculated from the power functions fitted to the measured data with R^2^ > 0.98.

After the measurements of odor threshold values, the exposure concentration as a function of dilution step was established for dilution steps with a detectable signal relative to the background noise of the olfactometer, see Figure 1. The best relationships between measured concentration at the nose cone outlet and dilution step was achieved by using power functions. The established functions were used to estimate the odorant exposure concentrations at dilutions steps below the detectable level. This extrapolation was necessary for all odorants except for dimethyl sulfide and is assessed to be a relatively accurate due to the high degree of correlation even in the low ppb-range, as seen in Figure 1. By using this approach, any loss or delayed breakthrough at the nose cone is corrected. For hydrogen sulfide, methanethiol, acetic acid, butanoic acid and 4-methylphenol, the nose cone concentrations were observed to be below the nominal concentrations (as calculated from the source concentration and the dilution factor) by a factor of ~2–6, corresponding to olfactometer recoveries in the range of 15 to 60%. Dimethyl sulfide, on the other hand, had an average recovery in the olfactometer above 95%, demonstrating the inertness of this compound. These observations (data not shown) are in line with recent results on olfactometer recoveries [6,7].

### 3.1. Selection of Panelists

A group of eight panelists was selected for the odor threshold measurements according to the European standard for dynamic olfactometry [1]. During the odor threshold measurements, the sensitivity of two panelists towards n-butanol decreased and as a result, they were outside the interval required by the standard. However, these two panelists were kept in the panel to see how the odor thresholds of other odorants were affected by panelists with lower sensitivity towards n-butanol. In Table 1 the average n-butanol threshold for each panelist is shown along with the average for the whole panel if either eight or six panelists are included. It is clear from Table 1 that the n-butanol threshold is lowered when the two outlying panelists are excluded. A statistical analysis revealed that when eight panelists were included there was a significant difference (*p* < 0.0001) between the panelists whereas with six panelists there is no significant difference (*p* = 0.16) between the panelists. 

### 3.2. Measured Odor Threshold Values

The odor threshold values of the six odorants included in the present study were estimated based on both eight and six panelists and are presented in Table 2. It is seen from Table 2 that whether eight or six panelists are included mainly has an effect on the threshold values for hydrogen sulfide, methanethiol and dimethyl sulfide. When the two outlying panelists (in relation to n-butanol) are excluded from the calculations the threshold values for hydrogen sulfide and methanethiol are lowered and the odor threshold value for dimethyl sulfide is increased whereas the threshold values for the other odorants are only slightly affected. There was a significant difference (*p* < 0.05) between the panelists for all six odorants and even when the two outlying panelists (in relation to n-butanol) are excluded there is still a significant difference (*p* < 0.05). This indicates that even though panelists are selected for n-butanol they do not necessarily have the same response towards other odorants. In the study by Klarenbeek et al. [30] it was shown that the panelists response towards n-butanol was not transferable to the response towards non-butanol measurements (single compounds and different waste gasses). Therefore, the threshold values from the present study that are included in the overall list of key odorants are based on all eight panelists. 

### 3.3. List of Key Odorants

The results from the present study are combined with literature values from other recent studies where the exposure concentrations were measured and a list of odor threshold values for 16 key odorants in pig house air is presented in Table 3. Only one of the studies included n-butanol [28] and none of the studies used n-butanol to select panelists. However, Nagata [28] determined an average odor threshold value for n-butanol of 38 ppb_v_, which is within the interval required in the European standard for dynamic olfactometry [1]. A number of the studies used olfactometers to estimate the threshold values [23,24,26,27,29], a few used high resolution gas chromatography-olfactometry (HRGC/O) [25,31] and one study used the triangle bag method [28]. In some cases only one reported study could be found where actual exposure concentration was measured (e.g., propanoic acid and 2-methylpropanoic acid) and in some cases the variation between reported values was large (e.g., acetic acid and 4-ethylphenol). Odor threshold values for a range of carboxylic acids were also reported by Punter [32]. However, the reported odor threshold values in that study were up to 60 times higher than the values reported in the present study. In the study by Punter [32] the concentrations at the different dilution steps were determined based on the input concentration to the olfactometer and the dilution factor based on a calibration with acetone. It has previously been demonstrated that an olfactometer can have a substantial influence on the recovery of odorants [6,7] and it is likely that the concentrations of odorants at the individual dilution steps were overestimated. Often when odor threshold values have been used to calculate odor activity values [10,22] for pig house air it has been based on the geometric mean of detection threshold values reported in the compilation by van Gemert [18]. In relation to sulfur compounds, there is a reasonable compliance between the threshold values estimated in the present study and the values previously used [10,22]. However, the present study and more recent studies confirm [23,25,26,27,28,29] that the odor threshold values of carboxylic acids (e.g., acetic and butanoic acid) might be lower. Particularly acetic acid is shown to have a threshold value that is ca. 30 times lower than the value previously used [10,22]. Furthermore, the present study and other studies [24,25,28,31] demonstrate that 4-methylphenol has a threshold value that is ca. 15 times lower than the value previously used [10,22]. The threshold value of trimethylamine reported in Table 3 is based on two studies [24,28] and the OTV is ca. 30 times lower than the value previously used [10,22]. Other studies concerned with trimethylamine have based their threshold measurements on solutions and headspace concentrations estimated by the Henry law constant [33,34] or the expected concentration after dilution in an olfactometer [35]. It has previously been demonstrated that trimethylamine has a very low breakthrough in an olfactometer probably due to surface adsorption [6,7] and it is very likely that the previous reported threshold values for trimethylamine were overestimated. Recent studies with 3-methylindole (skatole) [24,25,28] also show that the threshold value is ca. 30 times lower than the value previously used [10,22].

However, in order to use the threshold values for odor measurement or evaluation of odor abatement technologies, more information is needed on the potential combinatory effects of odorant mixtures. In principle, odorants could also be independent (meaning that no odor is perceived unless one of the odorants is above their odor threshold), antagonistic or synergistic. To our knowledge, no studies have been concerned with interaction between odorants from agricultural facilities. In the study by Wise et al. [29] they found that the interaction between carboxylic acids with small difference in carbon length (acetic acid and butanoic acid) was only additive at concentrations well below the threshold, whereas the interaction between carboxylic acids with larger difference in carbon length (e.g., acetic acid and hexanoic acid) was additive at all concentration levels. In the study by Miyazawa et al. [27] the interaction between carboxylic acids (acetic, butanoic, hexanoic, and octanoic acid) and structurally different odorants ((3-methyl-3-sulfanylbutyl) acetate, furan-2-ylmethanethiol, and 2-hydroxy-3-methylcyclopent-2-en-1-one) was investigated. In that study it was found that at concentrations well below the threshold of acetic acid and butanoic acid the interaction with the other odorants was additive, but at concentrations closer to the threshold it was subadditive, whereas for the other two carboxylic acids the interaction was subadditive at all concentration levels. These findings indicates that for short-chain acids (e.g., acetic and butanoic acid), which are normally dominating in air from pig houses, the interaction with some other odorants at a concentration level well below the threshold level the interaction is additive. A previous study [16] indicate that although complete additivity is not sufficient to estimate absolute odor concentration, some degree of additivity must exist. The “Equivalent odor concentration” approach presented by Wu et al. [16], which takes odor intensity and odor concentration into account shows promise in estimating the odor concentration. However, further investigations are needed to assess the generality of this. 

## 4. Conclusions

In conclusion, the analytical results of the present study shows that acetic acid, butanoic acid, and 4-methylphenol have significantly lower odor thresholds than previously estimated, which will have consequences for the assessment of odor from livestock production and the effects of odor abatement technology. Our study confirms previously reported odor threshold values for sulfur compounds (hydrogen sulfide, methanethiol and dimethyl sulfide). Consequently, acetic acid, butanoic acid, and 4-methylphenol may have a larger influence on odor from pig production facilities than previously assumed. However, more research is needed to investigate how odorants found in air from pig houses (e.g., sulfur compounds, carboxylic acids amines, phenols, and indoles) interact with both functionally related and none related odorants.

## Figures and Tables

**Table 1:** Individual odor threshold values (OTV) for n-butanol for the eight panelists included in the study and the average for all eight panelists or panelist 1–6 that fulfilled the requirements of the European standard for olfactometry.

Subject	N	OTV ^1^ Log(ppb_v_) ± SD ^2^	OTV ppb_v_
Panelist 1	54	1.72 ± 0.44	52
Panelist 2	54	1.71 ± 0.34	51
Panelist 3	54	1.59 ± 0.36	39
Panelist 4	54	1.58 ± 0.30	38
Panelist 5	54	1.66 ± 0.34	46
Panelist 6	54	1.70 ± 0.34	50
Panelist 7	54	2.31 ± 0.30	204
Panelist 8	53	2.21 ± 0.41	162
Average panelist 1–8	431	1.81 ± 0.44	65
Average panelist 1–6	324	1.66 ± 0.36	46

^1^ OTV: Odor threshold values; ^2^ SD: Standard deviation.

**Table 2:** Odor threshold values (OTV) based on eight panelists or the six panelists that fulfilled the requirements of the European standard for olfactometry.

**Panelists 1–8**	**N**	**OTV ^1^ Log(ppb_v_) ± SD ^2^**	**OTV ppb_v_**
Hydrogen sulfide	96	−0.38 ± 0.44	0.42
Methanethiol	94	−1.68 ± 0.51	0.021
Dimethyl sulfide	96	0.26 ± 0.28	1.8
Acetic acid	62	0.001 ± 0.22	1.0
Butanoic acid	62	−0.87 ± 0.39	0.14
4-methylphenol	63	−1.90 ± 0.50	0.013
**Panelists 1–6**			
Hydrogen sulfide	72	−0.44 ± 0.40	0.36
Methanethiol	70	−1.86 ± 0.35	0.014
Dimethyl sulfide	72	0.30 ± 0.29	2.0
Acetic acid	48	−0.01 ± 0.23	0.97
Butanoic acid	46	−0.88 ± 0.34	0.13
4-methylphenol	47	−1.89 ± 0.33	0.013

^1^ OTV: Odor threshold values; ^2^ SD: Standard deviation.

**Table 3:** A list odor threshold values (OTV, ppb_v_) for key odorants in pig house air based on recent literature values and results from the present study.

Odorants	Mean ^1^	[23]	[24]	[25] ^2^	[26]	[27]	[28]	[29]	[31]
Hydrogen sulfide	0.8			2.6			0.41		
Methanethiol	0.03		0.02				0.07		
Dimethyl sulfide	2.3						3		
Acetic acid	8.3	60		79	5.2	8.1	6	2.2	
Propanoic acid	5.7						5.7		
Butanoic acid	0.23		0.25	0.79	0.26	0.24	0.19	0.10	
Pentanoic acid	0.2			1.1			0.037		
2-methylpropanoic acid	1.5						1.5		
3-methylbutanoic acid	0.09			0.11			0.078		
Trimethylamine	0.08		0.21				0.032		
2,3-butandione	0.06			0.07			0.05		
Phenol	8.4						5.6		12
4-methylphenol	0.02		0.005	0.03			0.054		0.03
4-ethylphenol	0.4			0.09					1.5
Indole	0.06		0.013				0.3		
3-methylindole	0.003		0.001	0.004			0.0056		

^1^ Geometric mean of all references including the data from present study based on eight panelists (Table 2); ^2^ Geometric mean of reported min-max values except for hydrogen sulfide which was reported as one value.

## Supplementary Materials

The supplementary materials are available online at http://www.mdpi.com/1424-8220/18/3/788/s1.
