# Supplementary material for: Key Odorants from Pig Production Based on Improved Measurements of Odor Threshold Values Combining Olfactometry and Proton-Transfer-Reaction Mass Spectrometry (PTR-MS)
Source: Sensors (Basel). 2018 Mar 6;18(3):788. doi: 10.3390/s18030788 (PMC5876742; doi:10.3390/s18030788)
Supplement: Supplementary file 1 [file sensors-18-00788-s001.docx]

Key Odorants from Pig Production Based on Improved Measurements of Odor Threshold Values Combining Olfactometry and Proton-Transfer-Reaction Mass Spectrometry (PTR-MS)

Michael Jørgen Hansen ^1,^*, Pernille Lund Kasper ^1^, Anders Peter S. Adamsen ^2^ and
Anders Feilberg ^1^

^1^ Department of Engineering, Aarhus University, 8000 Aarhus, Denmark; peka@eng.au.dk (P.L.K.);
af@eng.au.dk (A.F.)

^2^ SEGES, 8200 Aarhus N, Denmark; apa@seges.dk

***** Correspondence: michaelj.hansen@eng.au.dk; Tel.: +45-2162-2710

Received: 22 December 2017; Accepted: 03 March 2018; Published: date

**Table A1.**  Detection limit for the calibration standard used for odorants included in the study

| **Compound** | **m/z** | **Detection limit, ppb_v_** |
| --- | --- | --- |
| Hydrogen sulfide | 35 | 0.45 |
| Methanethiol | 49 | 0.07 |
| Dimethyl sulfide | 63 | 0.11 |
| Acetic acid | 43 + 61 | 0.13 |
| Butanoic acid | 71 + 89 | 0.20 |
| 4-methylphenol | 109 | 0.23 |

**Table A2.**  Concentrations and relative standard deviation for the gas standards used in the study

| **Compound** | **m/z** | **Concentration, ppb_v_** | **Relative standard deviation for calibration standard, %** |
| --- | --- | --- | --- |
| Hydrogen sulfide | 35 | 5000 | ± 10 |
| Methanethiol | 49 | 5150 | ± 10 |
| Dimethyl sulfide | 63 | 5430 | ± 10 |
| Acetic acid | 43 + 61 | 4439 | ± 7 |
| Butanoic acid | 77 + 89 | 3212 | ± 7 |
| 4-methylphenol | 109 | 282 | ± 5 |
